# Characterization of Leptin and Leptin Receptor Gene in the Siberian Sturgeon (*Acipenser baerii*): Molecular Cloning, Tissue Distribution, and Its Involvement in Feeding Regulation

**DOI:** 10.3390/ijms26051968

**Published:** 2025-02-25

**Authors:** Hongwei Wu, Jiamei Li, Kezhen Jiang, Yingzi Li, Zhaoxiong Yu, Bin Wang, Bo Zhou, Xin Zhang, Ni Tang, Zhiqiong Li

**Affiliations:** 1 Department of Aquaculture, College of Animal Science and Technology, Sichuan Agricultural University, No. 211 Huimin Road, Chengdu 611130, China; whw409@163.com (H.W.);; 2 Fisheries Research Institute, Sichuan Academy of Agricultural Sciences, Chengdu 611731, China

**Keywords:** food intake, leptin, appetite regulation, appetite, fasting

## Abstract

Leptin is an adipokine known as a regulator of feeding and metabolism in mammals. Previous studies on fish have revealed its role in food intake regulation in limited teleosts. However, its specific function in Siberian sturgeon, an ancient Chondrostei fish, remains poorly understood. This study represents the first successful cloning of sequences for *leptin* and *leptin receptors* in Siberian sturgeon, achieved using RT-PCR. The predicted leptin sequence in this species consists of 168 amino acids that exhibit low identity with other fish species, except within the Acipenseriformes order. Tissue distribution analysis revealed a high expression of Siberian sturgeon *leptin* mRNA in the liver and *lepr* mRNA in the hypothalamus. Fasting differentially affected the expression of *leptin* and *lepr* mRNA, with decreased levels in the hypothalamus and increased levels in the liver (*leptin*: 3–15 days; *lepr*: 6–15 days). Recombinant Siberian sturgeon leptin (Ssleptin) was produced via *E. coli* expression, and intraperitoneal injection (100 ng/g BW) significantly inhibited food intake. The anorectic effect was correlated with changes in hypothalamic gene expression, including downregulation of orexigenic factors (*agrp*, *orexin*, *npy*, and *ghrelin*) and upregulation of anorexigenic factors (*pomc*, *mch*, and *insulin*). Meanwhile, the peripheral administration of Ssleptin promoted the expression of *resistin* in the liver and concurrently increased *cck* and *pyy* mRNA levels in the valvular intestine. Furthermore, Ssleptin injection stimulated the expression of hypothalamic *lepr*, *jak2*, *akt*, and *ampkα2* mRNA. These findings suggest that leptin plays a significant role in the feeding control of Siberian sturgeon and provide new insights into the evolutionary function of leptin in fish.

## 1. Introduction

Leptin as a hormone protein was originally discovered in mice [1]. It plays a role in various biological functions, including the regulation of food intake, energy homeostasis, lipid metabolism, and reproduction. Unlike mammals, which have only one identified type of *leptin* gene, multiple *leptin* genes and homologs have been detected in various fish species, likely due to gene duplications [2]. For example, one type of *leptin* has been identified in turbot (*Scophthalmus maximus*) [3], yellow catfish (*Pelteobagrus fulvidraco*) [4], and rainbow trout (*Oncorhynchus mykiss*) [5]. Two types of leptin genes have been identified in mandarin fish (*Siniperca chuatsi*) [6], tilapia (*Oreochromis niloticus*) [7], zebrafish (*Danio rerio*) [8], tongue sole (*Cynoglossus semilaevis*) [9], and orange-spotted grouper (*Epinephelus coioides*) [10]. Furthermore, two *leptin* homologs have been cloned from Atlantic salmon (*Salmo salar* L.) [11] and common carp (*Cyprinus carpio*) [12]. The *leptin* gene is highly conserved among mammals, with an 83% amino acid similarity observed between humans, mice, and rats. However, the similarity of leptin amino acids in teleost fish species is comparatively low. These findings suggest that the role of leptin in fish may be more complex and diversified compared to mammals.

Over the past few decades, several studies in teleosts have shown that leptin participates in feeding control but its effects on food intake remain controversial. Studies on rainbow trout [5], goldfish [13], and mandarin fish [6] have shown that the intraperitoneal (i.p.) injection of species-specific leptin significantly inhibited food intake. However, other studies on goldfish have reported that central treatment with human or mouse leptin protein did not affect food intake [14,15], which might be attributed to the structural differences in leptin proteins between teleost fishes and mammals. Therefore, it is crucial to study the function of leptin in fish using homologous leptin protein. To date, the mechanisms by which leptin regulates food intake in fish are not well understood.

Sturgeons have become one of the most popular aquaculture fish because of their protein-rich meat and caviar [16]. Owing to their pathogen resistance and high growth rate, Siberian sturgeon (*Acipenser baerii* brandt) is widely farmed worldwide [17]. In recent years, several studies have identified appetite-related factors in Siberian sturgeon, such as *npy*, *cart*, *resistin*, *galanin*, *apelin*, *nucb2*, *cck*, and *pyy* [18,19,20]. However, information about leptin in Siberian sturgeon remains largely unknown. This study aimed to reveal the function of Siberian sturgeon leptin in feeding regulation. Firstly, we cloned *leptin* and *leptin receptor* cDNA sequences and examined their expression in different tissues. The effects of fasting and refeeding on leptin signaling in the liver and hypothalamus were measured. Furthermore, the recombinant Siberian sturgeon leptin (Ssleptin) was expressed in *E. coli*, and the effect of Ssleptin on food intake and the expression of appetite-related factors in the hypothalamus, liver, and valvular intestine of Siberian sturgeon were investigated. Understanding the function of Siberian sturgeon leptin would provide significant insight into the early evolutionary history of leptin among vertebrates.

## 2. Results

### 2.1. Analysis of Leptin Sequence

**Figure 1:**
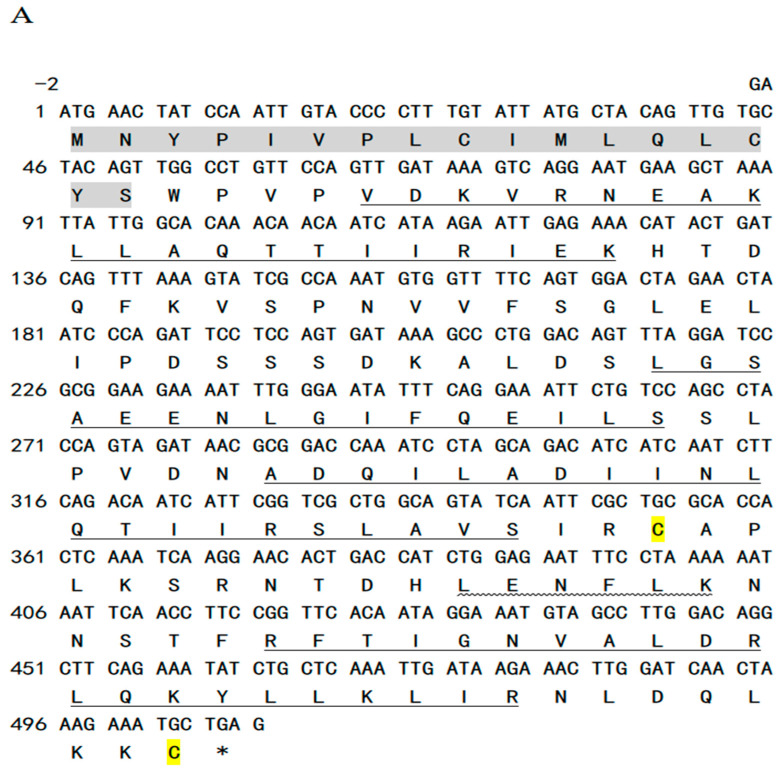

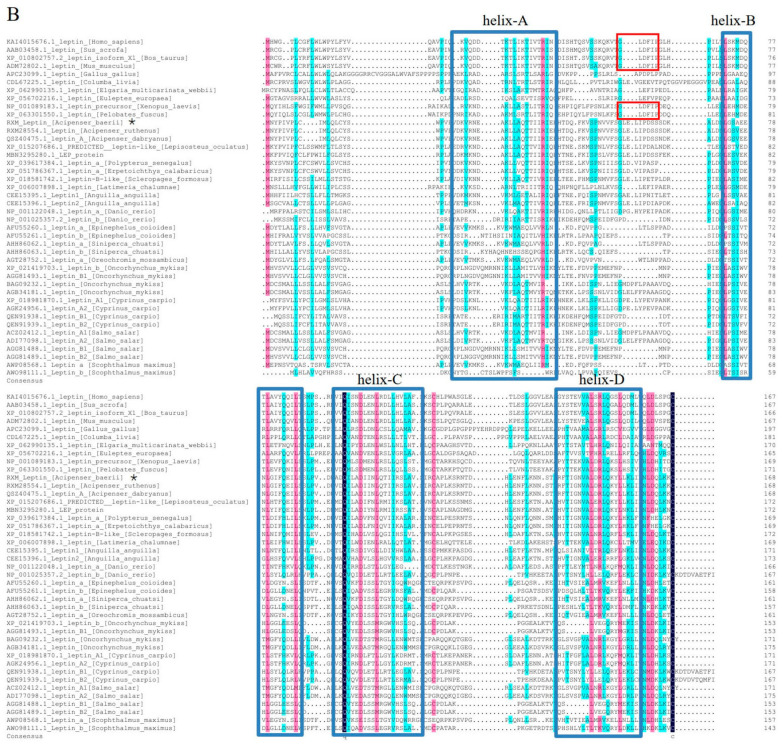

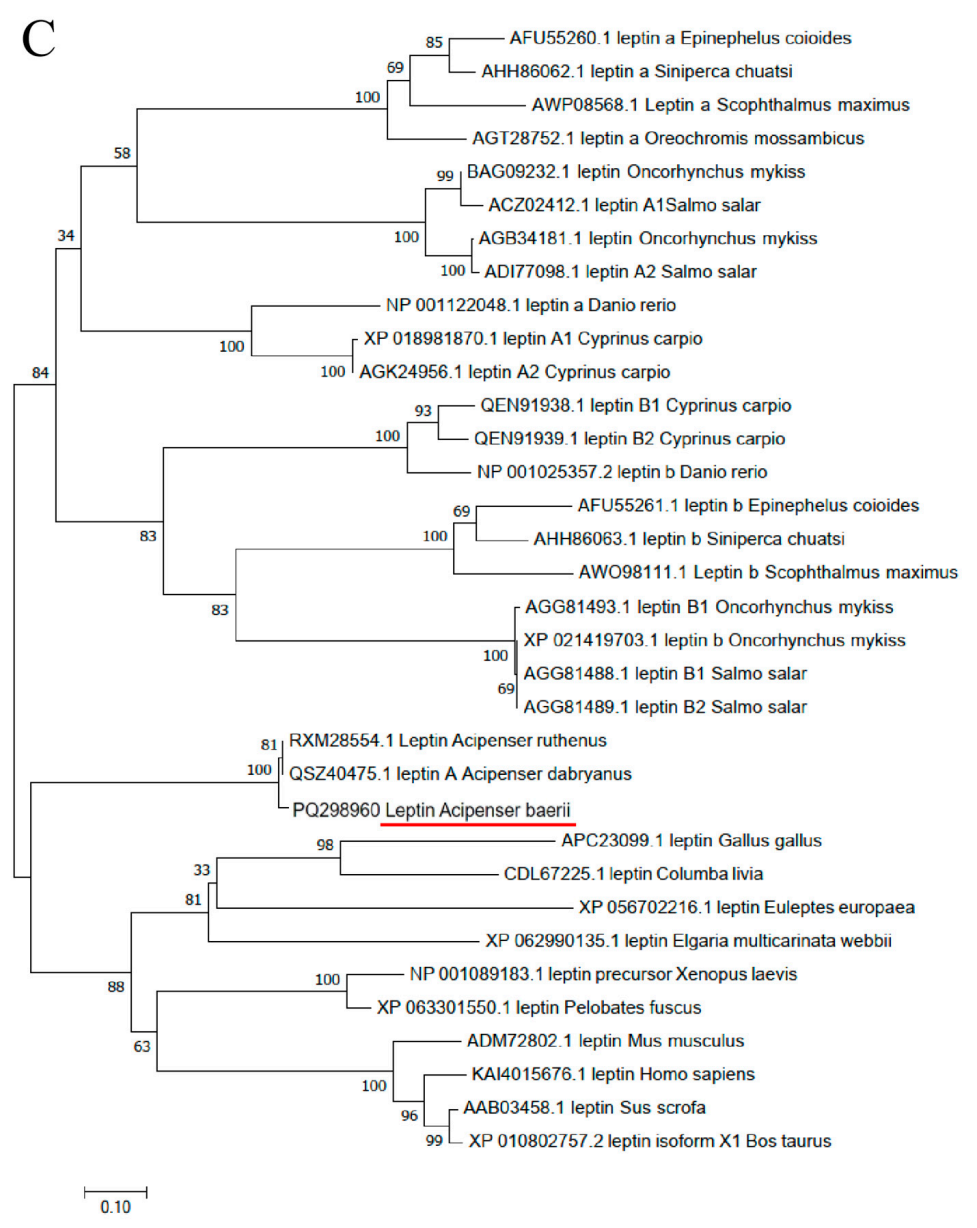
Sequence analysis of leptin in Siberian sturgeon. (**A**) Deduced amino acid sequence of the cloned leptin sequence. The signal peptide region was in gray; the underline represents helix A–D, the wavy line represents helix E, yellow means cysteine, and asterisk represents the termination codon. (**B**) Multiple-sequence alignment of amino acids. The asterisk means Siberian sturgeon leptin, the red box means the GLDFIP motif region, black highlight represents 100% amino acid consistency, red highlight represents ≥75, and sky blue highlight represents ≥50. (**C**) Phylogenetic analysis of leptin; red line: Siberian sturgeon.

This study successfully cloned the CDS sequence of *leptin* and a portion of the leptin receptor’s CDS fragment (Figure S1) using RT-PCR. The cloned *leptin* cDNA in Siberian sturgeon was 510 bp, encompassing a 507 bp ORF, which encoded a precursor protein of 168 amino acids. Siberian sturgeon leptin contained a signal peptide (1–17 aa) and alpha-helical domains (designated helix A–D, located at amino acid positions 23–41, 73–87, 93–115, and 143–164). Furthermore, a small helical segment was identified between helix C and D (helix E, 129–136 aa). In addition, the leptin protein in Siberian sturgeon featured a pair of cysteine residues at positions 118 and 168 (Figure 1A).

Siberian sturgeon leptin precursor protein showed high similarity to those of *Acipenser ruthenus* (98.80%) and *Acipenser dabryanus* (98.21%). However, the sequence identity between Siberian sturgeon leptin and other species was relatively low, ranging from 14.2% to 64.8%. Specifically, the identity of fish species (*Lepisosteus_oculatus*, 64.28%; *Amia calva*, 64.88%; *Polypterus senegalus*, 56.54%; *Erpetoichthys calabaricus*, 55.35%; *Scleropages formosus*, 53.57%; *Latimeria chalumnae*, 39.28%; *Anguilla_anguilla* leptin1, 44.64%; *Anguilla_anguilla* leptin2, 46.42%; *Danio_rerio* leptinA, 28.31%; *Danio_rerio* leptinB, 22.02%; *Epinephelus_coioides* leptinA, 17.39%; *Epinephelus_coioides* leptinB, 19.62%; *Siniperca chuatsi* leptinA, 19.25%; *Siniperca chuatsi* leptinB, 19.62%; *Oreochromis mossambicus* leptinA, 19.88%; *Oncorhynchus mykiss* leptinB, 20.26%; *Oncorhynchus mykiss* leptinB1, 17.64%; *Oncorhynchus mykiss* leptin, 23.81%; *Oncorhynchus mykiss* leptin, 25.00%; *Cyprinus_carpio* leptinA1, 36.30%; *Cyprinus_carpio* leptinA2, 36.90%; *Cyprinus_carpio* leptinB1, 20.24%; *Cyprinus_carpio* leptinB2, 20.83%; *Salmo salar* leptinA1, 23.21%; *Salmo salar* leptinA2, 24.40%; *Salmo salar* leptinB1, 19.61%; *Salmo salar* leptinB2, 19.61%; *Scophthalmus maximus* leptinA, 18.47%; *Scophthalmus maximus* leptinB, 18.88%), amphibians (*Xenopus laevis*, 35.11%; *Pelobates fuscus*, 35.11%), reptiles (*Elgaria multicarinata webbii*, 22.02%; *Euleptes europaea*, 18.78%), birds (*Gallus gallus*, 14.28%; *Columba livia*, 16.66%), and mammals (*Homo sapiens*, 25.74%; *Sus scora*, 28.74%; *Bos taurus*, 28.31%; *Mus musculus*, 25.74%) were low (Figure 1B). Phylogenetic analysis revealed that Siberian sturgeon leptin clustered closely with leptin from *Acipenser ruthenus*, *Acipenser dabryanus*, *Polypterus senegalus*, *Erpetoichthys calabaricus*, *Amia calva,* and *Lepisosteus oculatus* (Figure 1C).

### 2.2. Tissue Distribution of Leptin and Lepr mRNA in Different Tissues of Juvenile Siberian Sturgeon

**Figure 2:**
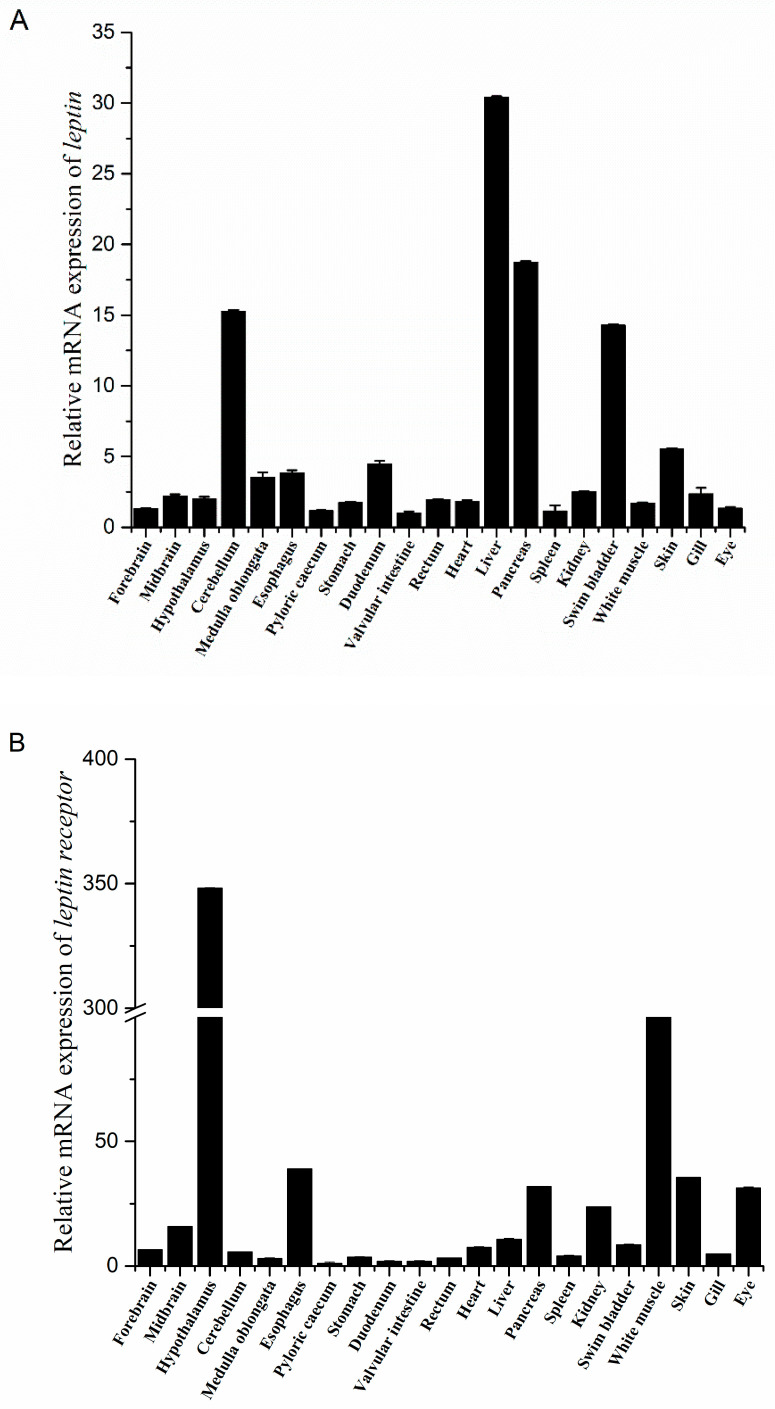
Tissues distribution of leptin (**A**) and leptin receptor (**B**) mRNA.

*Leptin* and its receptor (*lepr*) were widely expressed in both the peripheral tissues and the brain of juvenile Siberian sturgeon. As shown in Figure 2A, The highest expression of *leptin* mRNA was found in the liver, followed by the pancreas, cerebellum, and swim bladder (Figure 2A). An abundant expression of *lepr* mRNA was observed in the hypothalamus and white muscle, with notable levels in the esophagus, skin, eyes, pancreas, and kidneys and low expression in the pyloric caecum, duodenum, and valvular intestine (Figure 2B).

### 2.3. Relationship Between Leptin Systerm and Feeding Status

**Figure 3:**
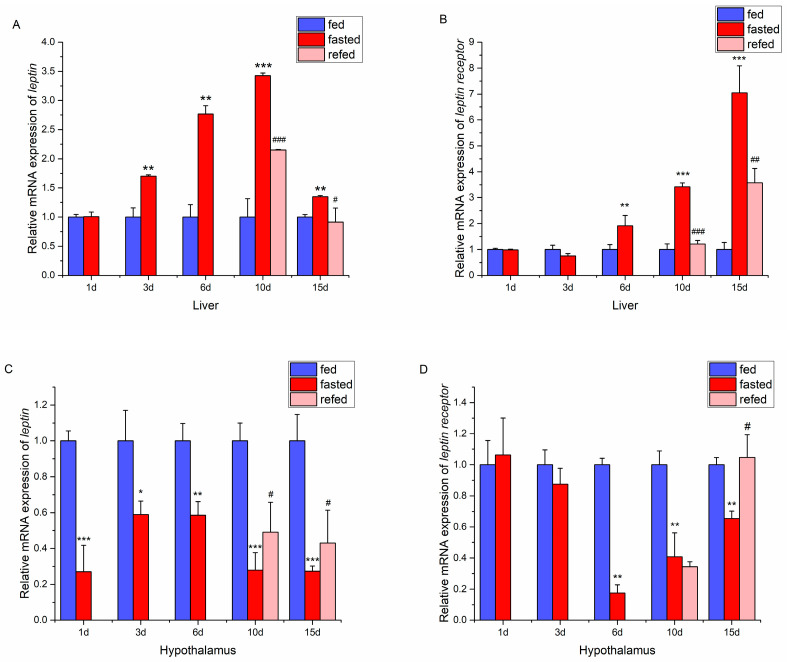
Relative mRNA expressions of leptin and leptin receptor under different nutritional statuses in the liver (**A**,**B**) and hypothalamus (**C**,**D**). The gene expression was normalized with β-actin. Student’s *t*-test was used to analyze the two groups’ differences at the same time point. * means *p* < 0.05 between feeding group and fasted group, ** means *p* < 0.01 between feeding group and fasted group, *** means *p* < 0.001 between feeding group and fasted group; # means *p* < 0.05 between fasted group and refeeding group, ## means *p* < 0.01 between fasted group and refeeding group, ### means *p* < 0.001 between fasted group and refeeding group.

Considering that leptin and leptin receptors were abundantly expressed in the liver or hypothalamus, this study explored whether fasting affected the liver and hypothalamic *leptin* and *lepr* expression in juvenile Siberian sturgeon. It was noted that the mRNA expression of *leptin* in the liver of juvenile Siberian sturgeon in the fasting group was higher than in the fed control group on days 3, 6, 10, and 15 (*p* < 0.01, Figure 3A). Similarly, liver *lepr* mRNA expression in the fasted groups was higher than in the fed control group on days 6, 10, and 15 (*p* < 0.01, *p* < 0.001, *p* < 0.001, Figure 3B). The increased expression of *leptin* and its receptor mRNAs induced by 10 days of fasting was significantly reduced after refeeding for 1 or 5 days.

In the hypothalamus, *leptin* expression in the fasting group was lower than that in the feeding group during the 1–15 day period (Figure 3C). In addition, the mRNA expression of *lepr* in the fasting group on days 6, 10, and 15 was lower than that in the feeding group (Figure 3D). Refeeding after 10 days of fasting promoted the mRNA expression of *leptin* in the hypothalamus. However, the decrease of hypothalamic *lepr* mRNA expression induced by fasting was not immediately reversed after 1 day of refeeding but showed significant recovery after 5 days of refeeding.

### 2.4. Recombinant Ssleptin Expression

**Figure 4:**
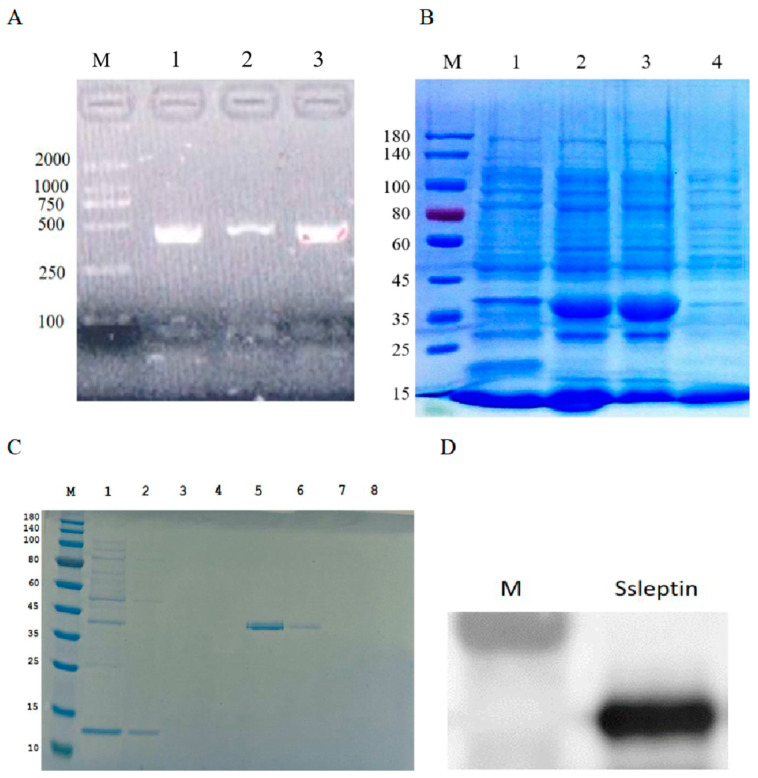
(**A**) The PCR amplification of leptin sequence using agarose gel electrophoresis analysis. M: maker, lane 1–3: the PCR product of leptin mature domain. (**B**) The soluble examination of Ssleptin protein using SDS-PAGE electrophoresis analysis: 1: vector protein samples induced for 6 h; 2: Ssleptin samples induced for 6 h; 3: the sediment of SsLeptin induced for 6 h and then lysed; 4: the supernatant of Ssleptin induced for 6 h and then lysed. (**C**) Different doses of imidazole eluted target protein using SDS-PAGE electrophoresis analysis. 1: Protein efflux: 2–8: different imidazole concentrations (10, 20, 50, 100, 150, 200, 300 mM). (**D**) Western blot analysis about Ssleptin.

To obtain the Ssleptin recombinant protein, the mature domain of leptin of 456 bp was successfully inserted into the expression vector (Figure 4A). The Ssleptin protein encoded a protein of 37.43 kDa containing 6 × His tags. The Ssleptin was highly expressed in the sediment after fragmentation with the form of inclusion bodies (Figure 4B). The target protein solution was purified with Ni-NTA Resin column; 8 M urea containing 10 mM imidazole, which can wash out a large amount of impurity proteins; and the 8 M urea buffer containing 100 mM imidazole, which could mainly elute the target protein (Figure 4C). The Ssleptin recombinant protein contained the His tags after Western blotting analysis (Figure 4D).

### 2.5. Effect of Ssleptin on Food Intake

**Figure 5:**
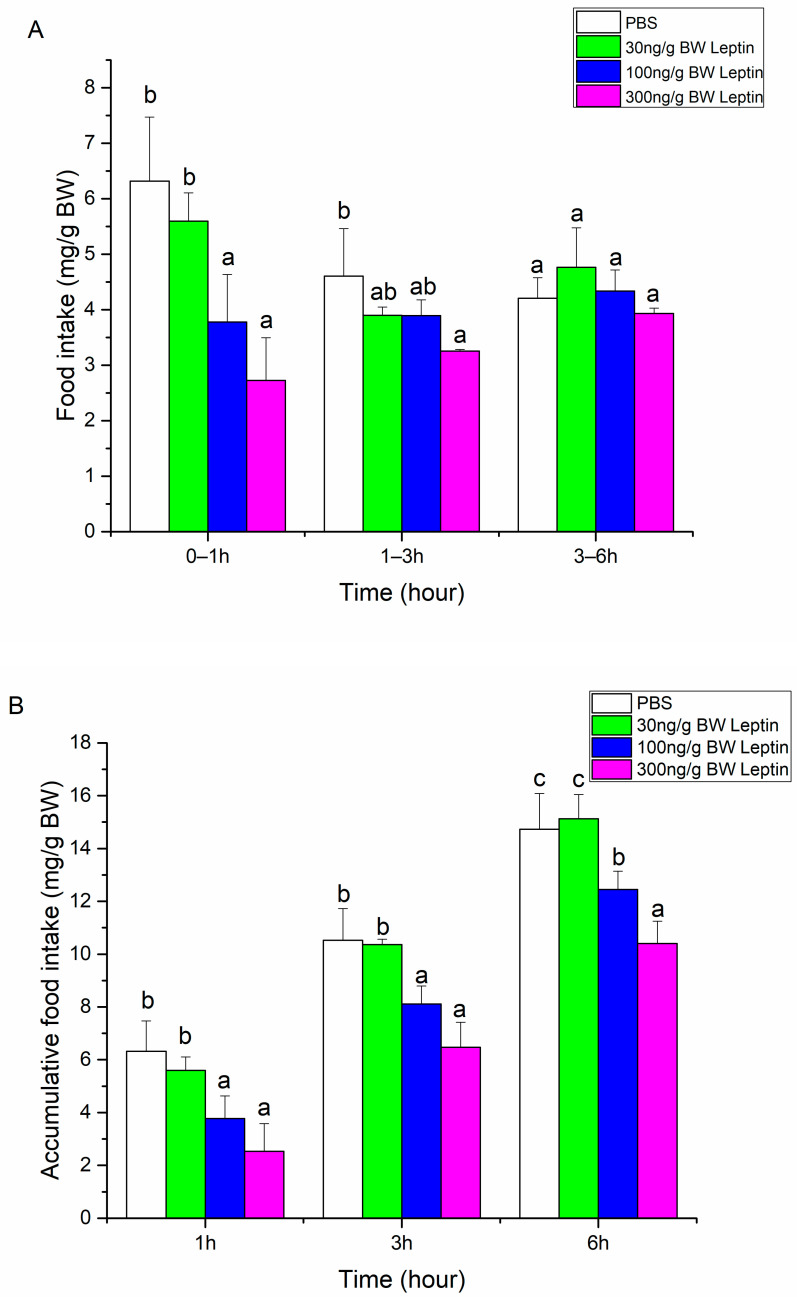
Effect of peripheral Ssleptin on food intake during period time (**A**) or accumulative time (**B**) of Siberian sturgeon. Different letters mean significant differences in food intake among groups (*p* < 0.05).

To evaluate whether Ssleptin could function as an anorexic factor in Siberian sturgeon, food intake was measured following the acute intraperitoneal administration of recombinant Ssleptin protein. Compared with the phosphate-buffered saline (PBS) group, food intake in the 0–1 h period was significantly reduced following the administration of 100 and 300 ng/g BW Ssleptin (*p* < 0.05), while 30 ng/g BW doses showed no significant effect during this time. Food intake during a 1–3 h period was inhibited after injection of 300 ng/g BW Ssleptin compared with PBS treatment (Figure 5A). When injected with 100 ng/g BW or 300 ng/g BW Ssleptin, the cumulative food intake at all tested points (1, 3, and 6 h) was significantly inhibited compared to PBS administration (*p* < 0.05) (Figure 5B).

### 2.6. Ssleptin Action on Genes Related to Feeding in Siberian Sturgeon

**Figure 6:**
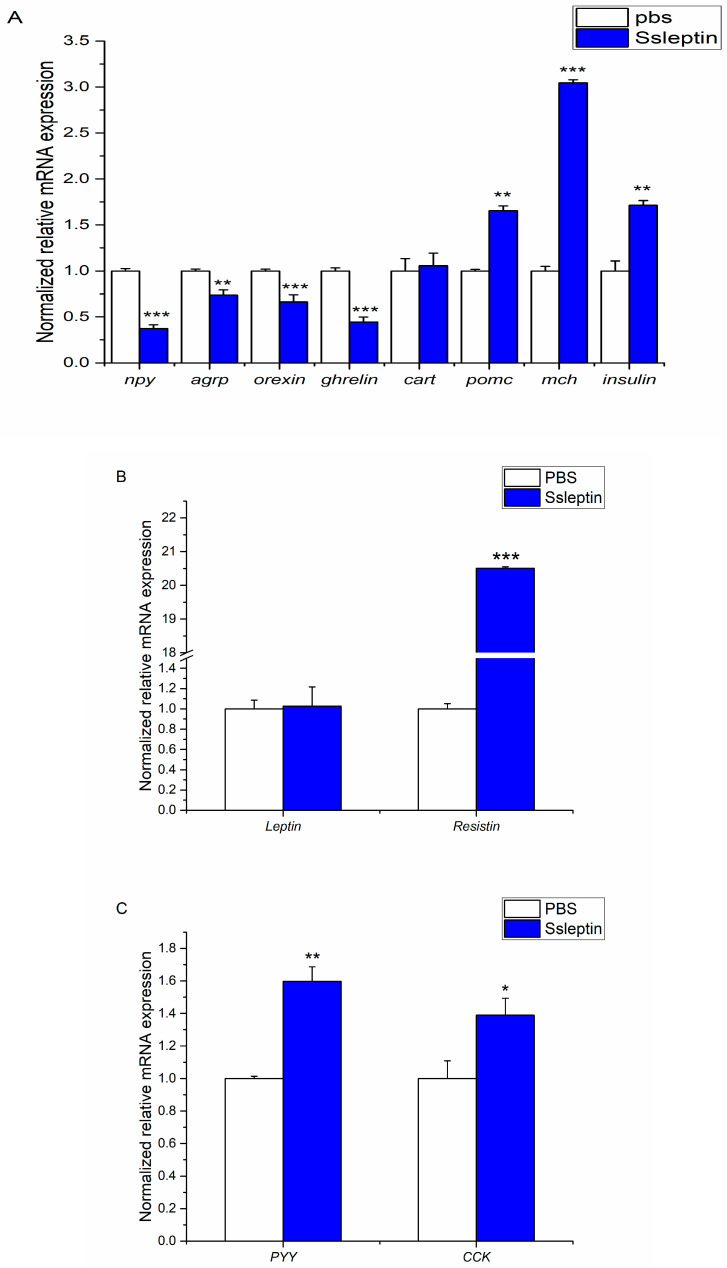
Effect of peripheral Ssleptin on appetite factors expression in the hypothalamus (**A**), liver (**B**), and valvular intestine (**C**). A comparison of the Ssleptin group and control group was analyzed with the *t* test. * means *p* < 0.05; ** means *p* < 0.01; *** means *p* < 0.001. Each column represents six fish (*n* = 6).

To evaluate the role of Ssleptin in appetite regulation, this present study examined the expression of appetite-related genes, including *agrp*, *npy*, *ghrelin*, *orexin*, *cart*, *pomc*, *mch*, *insulin*, *resistin*, *pyy*, and *cck* in the hypothalamus, liver, and valvular intestine after Ssleptin administration. At 1 h post-injection of Ssleptin (100 ng/g BW), the mRNA levels of *npy*, *agrp*, *orexin*, and *ghrelin* were significantly inhibited, while *pomc*, *mch*, and *insulin* were upregulated in the hypothalamus. In the liver, *resistin* expression was elevated after Ssleptin administration (*p* < 0.001). The mRNA expressions of *pyy* (*p* < 0.01) and *cck* (*p* < 0.05) in the valvular intestine were increased following SsLeptin injection (Figure 6).

### 2.7. Ssleptin Action on Signaling Pathway Genes Associated with Appetite

**Figure 7:**
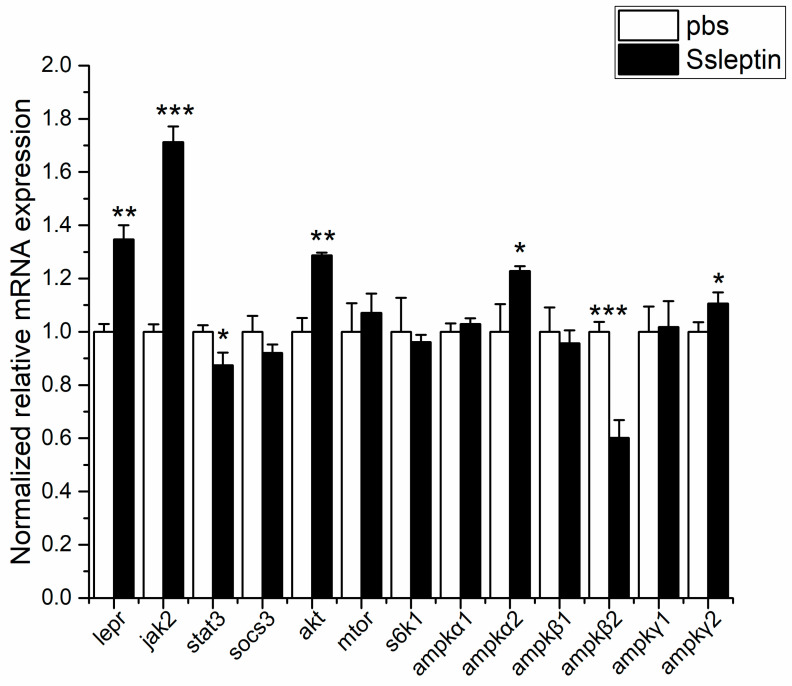
Effect of i.p. injection of Ssleptin on the mRNA expressions of signal pathway factors in hypothalamus of Siberian sturgeon. The data represent mean ± S.E.M (*n* = 6). A comparison of the PBS group with the Ssleptin group was analyzed using the *t* test, marking the significant levels with * (*p* < 0.05), ** (*p* < 0.01), *** (*p* < 0.001).

It was found that the mRNA levels of related factors in the JAK–STAT pathway were modulated after Ssleptin treatment, including the increase in *jak2* mRNA expression (*p* < 0.001) and the decrease in *stat3* expression (*p* < 0.05). The intraperitoneal injection of Ssleptin significantly increased *ampkα2* gene expression in *ampkα* subunits (*p* < 0.05) and decreased *ampkβ2* expression in *ampkβ* subunits (*p* < 0.001), with no significant changes in other subunit genes. In addition, *akt* expression was significantly increased after SsLeptin injection (*p* < 0.01), while *mtor* and *s6k1* mRNA expressions remained unchanged. Additionally, the expression of *lepr* was significantly increased following Ssleptin treatment (*p* < 0.01) (Figure 7).

## 3. Discussion

### 3.1. Siberian Sturgeon Leptin Structure

The cloned *leptin* sequence of the Siberian sturgeon analyzed in this study includes a signal peptide, four reverse helix domains, and one small helix segment (helix E) situated in the loop connecting helix C and helix D. This result aligns with most studies on the secondary structure of leptin in both mammals and fish [2]. Moreover, similar to other fish species such as the snakehead fish [21] and Yangtze sturgeon [22], we identified a pair of cysteine residues (located at positions 118 and 168 aa, respectively) in the leptin of the Siberian sturgeon, which are capable of forming disulfide bonds that connect helixes C and D. The spatial configuration of the four reverse helix domains, supported by these disulfide bonds and helix E, is essential for binding to leptin receptors. The GLDFIP motif within the leptin molecule is recognized as being crucial for activating the leptin receptor in mammals [21]. However, Siberian sturgeon leptin lacks the GLDFIP sequence, similar to other teleosts [23]. This difference may indicate an evolutionary adaptation leading to distinct leptin–receptor interactions in aquatic vertebrates. These results suggest that the molecular mechanisms governing leptin receptor binding and activation could diverge significantly between fish and mammals. Further studies are needed to explore the interaction between leptin and its receptor.

Unlike mammals, most bony fish species possess more than two types of leptin genes (*leptin-a* and *leptin-b*), as seen in species such as the mandarin fish [6], goldfish [13], and zebrafish [8], which have undergone additional whole genome duplications (WGD). Although we screened the transcriptome database of Siberian sturgeon liver, only one *leptin* sequence was found, consistent with previous studies on Yangtze sturgeon [22]. This finding may be attributed to the slow evolution rate of sturgeon, which prevented whole-genome replication events during the period of 350–400 Mya [22]. Phylogenetic analysis of the cloned sequence from this study revealed its close relationship with *leptin-a* in other fish species.

### 3.2. Tissue Distribution of Leptin and Leptin Receptor

Siberian sturgeon leptin mRNA was highly expressed in the liver, followed by the pancreas, cerebellum, and swim bladder, which was in line with Ya fish [24], pufferfish [25], carp [12], green catfish [26], and rainbow trout [5]. In mammals, leptin is mainly synthesized and secreted by white adipose tissue [27]. In this study, no significant adipose tissue was found in the juveniles of Siberian sturgeon. In addition, the tissue distribution of *leptin* in other teleost fishes varies significantly, with different subtypes exhibiting distinct expression patterns. For instance, *leptin* was abundantly expressed in the adipose tissue of some perciform fish like Nile tilapia [7] and mackerel [28], while in numerous teleost species, leptin was almost absent in adipose tissue [12,29,30,31,32]. This discrepancy may be attributed to the liver’s function as an important lipid storage site in fish. Conversely, high leptin expression was observed in the brain tissues of several fish species, including the spotted grouper [10], Atlantic salmon [11], and half-smooth tongue sole [9]. These differences between mammals and fish suggest that the site of leptin production has evolved over time.

In Siberian sturgeon, *leptin receptor* (*lepr*) was predominantly expressed in the hypothalamus, consistent with findings on goldfish [33]. However, tissue- and species-specific expression patterns of *lepr* have been reported in other teleost fish. For instance, *lepr* mRNA was abundant in the kidneys of orange-spotted grouper [10] and Atlantic salmon [34]. In Japanese medaka (*Oryzias latipes*), *lepr* was highly expressed in the muscles, skin, and gills [26], while in yellow catfish (*Pelteobagrus fulvidraco*), *lepr* was predominantly expressed in the pituitary [4]. Moreover, the expression of leptin receptor paralogs varied across tissues. For example, *lepra1* and *lepra2* mRNA in rainbow trout were abundantly expressed in the ovaries [35], whereas in Atlantic salmon, *lepra1* was highly expressed in the red muscle and brain and *lepra2* was highly expressed in the ovaries and liver [36]. The widespread tissue distribution of *lepr* in teleosts suggests multiple physiological functions, including roles in reproduction and metabolism. Interestingly, consistent with this study, the long-form leptin receptor was primarily expressed in the hypothalamus of mammals [37]. These observations suggest that lepr may play a role in the feeding regulation of Siberian sturgeon. This present study detected leptin and lepr expression in the juvenile stage. Whether their expression patterns are conserved across different life stages, such as larval, juvenile, and adult, needs to be further explored.

### 3.3. Fasting Influenced Leptin Expression in the Hypothalamus and Liver

In this study, increased leptin mRNA expression was observed in Siberian sturgeon livers after fasting for 3–10 days. Similar results were reported in orange-spotted grouper (leptin-a, fasting for 7 or 21 days) [10], adult male tilapia (*Oreochromis mossambicus*) [38], and yellow cheek carp (*Elopichthys bambusa*) (fasting for 8 days) [39]. However, in contrast to this study, several reports in zebrafish (fasting for 7 days) [8] and common carp (fasting for 6 or 42 days) [12] showed that liver *leptin-a* mRNA expression was unaffected. Moreover, fasting inhibited liver *leptin-b* expression in zebrafish [8] and *leptin* expression in striped bass [40]. Additionally, fasting for 1–15 days inhibited hypothalamic *leptin* expression in Siberian sturgeon. Similarly, fasting for 3 or 8 days significantly inhibited *leptin* expression in the brain of yellow cheek carp [39]. These results suggest that endogenous leptin in the hypothalamus of Siberian sturgeon may rapidly decrease in response to energy deficiency, while persistent energy deficiency might stimulate leptin production in the liver, thereby regulating energy homeostasis.

The mRNA expression of *lepr* in the liver and hypothalamus of Siberian sturgeon also changed in response to fasting. Although fasting for three days did not affect liver *lepr* expression, fasting for 6 days promoted *lepr* expression in Siberian sturgeon. Similarly, increased *lepra2* expression in the liver was observed in rainbow trout after fasting for several days (7, 14 days) but not affected when fasting for one day [35]. Hepatic *lepr* mRNA expression was also promoted after fasting for 7 days in tilapia [38]. Interestingly, the opposite effect of fasting on *lepr* expression was observed in the hypothalamus of Siberian sturgeon. These findings suggest that fasting could differentially affect the expressions of leptin and leptin receptors in the liver and hypothalamus of juvenile Siberian sturgeon. These results suggest that both leptin and leptin receptors might play important roles in the regulation of energy homeostasis.

### 3.4. Ssleptin Inhibited Food Intake of Siberian Sturgeon

Leptin is a protein hormone known to regulate energy balance and act as a satiety factor, reducing the food intake of animals [41]. Several studies have reported its regulatory effects on food intake in several bony fish species, including those from the Cypriniformes, Salmoniformes, and Perciformes orders. However, its role in Siberian sturgeon remains unexplored. This study reveals that the intraperitoneal injection of recombinant Siberian sturgeon leptin (Ssleptin) promptly inhibited food intake at 1 h after administration. This finding aligns with previous reports where leptin produced through prokaryotic expression systems suppressed food intake in rainbow trout [5], Nile tilapia [30], grass carp [42], goldfish [13], and mandarin fish [6]. In contrast, centrally administrated rat leptin did not affect the food intake of channel catfish [43], a result consistent with findings in goldfish treated with murine or human leptin [14,15]. These discrepancies may stem from the use of heterologous mammalian leptins, given the low identity between mammalian and fish leptins. Here, we demonstrate for the first time that peripheral Ssleptin can effectively inhibit feeding in Siberian sturgeon.

The observed changes in food intake were closely linked to the expression of appetite-regulating factors in the brain and peripheral tissues. The mechanism of leptin in feeding regulation in Siberian sturgeon was investigated by measuring the expression of several appetite factors in the hypothalamus, liver, and valvular intestine. In this study, after Ssleptin administration, the expression of *npy* and *agrp* was reduced in the hypothalamus of Siberian sturgeon, aligning with previous findings on rainbow trout [5], goldfish [13], and Nile tilapia [30]. At present, little is known about the relationship between leptin and other orexigenic factors. In addition, this study found that orexgenic factors, including *orexin* and *ghrelin*, were reduced in the hypothalamus of Siberian sturgeon upon intraperitoneal injection of Ssleptin. In zebrafish, it has been reported that knocking out the *leptin* gene increases *ghrelin* expression in the brain [44]. In addition, the i.p. injection of leptinAI and leptinAII inhibited *orexin* expression in the hypothalamus of goldfish [13]. A previous report on rats revealed that the increase in *orexin* expression induced by fasting in the hypothalamus could be inhibited by the intraventricular administration of leptin [45]. These observations indicate that the anorexigenic effect of leptin might be modulated by orexigenic factors (*npy*, *agrp*, *orexin*, and *ghrelin*), a mechanism that appears to be conserved among fish species and mammals.

POMC/CART neurons, which are classic anorexigenic neurons in the feeding regulation center, suppress appetite when activated [46]. Our study showed that the intraperitoneal injection of Ssleptin promoted the expression level of hypothalamic *pomc*, consistent with findings on rainbow trout [5] and goldfish [13]. The anorexigenic role of *cart* has been demonstrated in Siberian sturgeon [47], goldfish [14], Yafish [48], and Atlantic salmon [49]. A previous study on goldfish revealed that the i.p. injection of leptinA promoted hypothalamus *cart* expression [13]. However, in this study, hypothalamic *cart* expression in Siberian sturgeon was not changed by peripheral Ssleptin administration. Additionally, the MCH neuron situated in the hypothalamus is responsible for the production of MCH neuropeptides, which modulate feeding behavior [50]. In rats, the continuous intraventricular injection of leptin decreased body weight and food intake, concurrent with a decrease in hypothalamic *mch* expression [51]. Our study revealed that Ssleptin injection led to a significant increase in *mch* expression in the hypothalamus, suggesting a species-specific response. This difference could be attributed to the varying effects of MCH on appetite regulation across fish species and mammals. For example, the intraventricular injection of MCH significantly inhibited feeding in goldfish [52], while it had the opposite effect on mice [53]. Moreover, MCH neuron activation might enhance leptin’s access to the hypothalamus by promoting microvessel fenestration [54]. Ssleptin could promote insulin expression in the hypothalamus of Siberian sturgeon. This aligns with observations of rats, where insulin or leptin administrated singly or in combination suppressed feeding [55], and with the concept that hypothalamic *leptin* can enhance insulin sensitivity [56]. Collectively, these observations indicate that the intraperitoneal administration of Ssleptin acutely inhibits the feeding of Siberian sturgeon through the upregulation of anorexigenic factors, including *pomc*, *mch*, and *insulin* in the hypothalamus.

In the peripheral tissues, leptin and resistin are mainly produced by the fish liver, and peptides YY (PYY) and cholecystokinin (CCK) are mainly secreted by intestinal endocrine cells [2,57,58,59]. This study showed that the intraperitoneal injection of Ssleptin did not affect liver *leptin* expression but increased *resistin* expression. In rats, the central injection of resistin rapidly reduced food intake [60,61]. Both *pyy* as well as *cck* mRNA expressions in the valvular intestine of Siberian sturgeon were promoted by the intraperitoneal administration of Ssleptin. Both PYY and CCK are known to inhibit food intake in Siberian sturgeons [18,19]. Therefore, peripheral Ssleptin treatment can act in the valvular intestine to promote the expression of *pyy* and *cck* and in the liver to promote *resistin* expression, thereby exerting an inhibitory effect on the feeding of Siberian sturgeon. The interactions between leptin and these appetite-regulating hormones in Siberian sturgeon require further investigation using coinjection experiments or RNA interference to elucidate the complex regulatory network controlling feeding behavior.

### 3.5. Ssleptin Stimulated Lepr/JAK2/AKT/AMPKα2 in the Hypothalamus of Siberian Sturgeon

In fish, studies on the molecular mechanisms of leptin in feeding control are limited. Only a few reports on rainbow trout have reported the mechanism involved in leptin signaling [62]. This present study showed that *lepr* was abundantly expressed in the hypothalamus of Siberian sturgeon. The peripheral administration of Ssleptin stimulated *lepr* expression in the hypothalamus. Similarly, it has been reported that the activation of LepR neurons in the NTS decreased food intake and body weight in mice [63]. However, a previous study in adult male zebrafish showed that the mutation of *lepr* did not affect food intake or weight [64]. This difference may be attributed to the unique evolutionary position of Siberian sturgeon, which diverged from the actinopterygian lineage after the ancient 2R WGD event and before the teleost-specific 3R WGD event, whereas zebrafish experienced the 3R WGD event [65]. These observations suggest that the leptin receptor might be involved in mediating the anorexigenic role of Ssleptin in the feeding control of juvenile Siberian sturgeon.

In mammals, leptin is known to modulate the JAK–STAT, PI3K–AKT, mTOR, and AMPK signaling pathways, which regulate appetite [66]. Our results demonstrated that the intraperitoneal administration of Ssleptin could lead to the increase of *akt* mRNA levels in the hypothalamus of Siberian sturgeon. Similarly, an in vitro incubation assay revealed that leptin could elevate the phosphorylation level of AKT in the hypothalamic cells of rainbow trout [62]. The above observations suggest that leptin might activate AKT signaling to exert its anorexigenic effect in Siberian sturgeon. A recent study on mice has shown that the microinjection of leptin can activate JAK2 to inhibit feeding in the basolateral amygdala (BLA) neurons of the hypothalamus [67]. This is similar to this study, wherein peripheral Ssleptin administration increased hypothalamic *jak2* expression. However, we found a decrease in *stat3* mRNA levels in the hypothalamus induced by Ssleptin administration. In contrast, a study on rainbow trout has shown that leptin can promote STAT3 phosphorylation in hypothalamic cells [62]. This difference might be due to the fact that this study examined *stat3* transcript levels rather than phosphorylation levels, which needs further investigation. In addition, we observed that the expression of hypothalamic *ampkα2* was promoted after Ssleptin treatment but not *ampkα1*. Similarly, a study on rainbow trout showed that *ampkα2* but not *ampkα1* was involved in feeding regulation [68]. In rats, the inhibitory effect of leptin on feeding was eliminated after AMPKα2 siRNA administration [69]. These observations suggest that hypothalamic *jak2*, *akt*, and *ampkα2* might mediate the inhibitory effect of Ssleptin on food intake in Siberian sturgeon.

## 4. Materials and Methods

### 4.1. Experimental Animals

Juvenile Siberian sturgeon were obtained from Runzhao Fisheries (Chengdu, China). All fish were reared in 120 L tanks with aerated freshwater at a temperature of 20.2 ± 1.4 °C under natural light conditions. The fish were fed with commercial sturgeon feed (protein 42%, fat 6%, ash 16%, fiber 4%, moisture 12%, phosphorus 0.8%, lysine 1.8%; Tongyi, China) once daily at 14:00 for 14 days. The animal handling procedures were approved by the Animal Care and Use Committee of Sichuan Agricultural University (Approval Code: 20230081 and Approval Date: 8 March 2023).

### 4.2. Cloning of Siberian Sturgeon Leptin and Leptin Receptor and Sequence Analysis

Primers (Table 1) used for amplifying the *leptin* or *leptin receptor* coding regions were designed with Primer 6.0 based on the sequence obtained from the *Acipenser ruthenus* GenBank Database (GenBank XM_034032711.1, GenBank XM_034006887.2). Total RNA for cloning target genes originated from the hypothalamus and liver of juvenile Siberian sturgeon following the instructions of the total RNA extracted kit (Forgene, Chengdu, China). RNA (1 μg) was reverse-transcribed into cDNA using a two-step method according to the protocols of a cDNA synthesis kit (Takara, China) containing gDNA eraser to remove genomic DNA. The 2 × Taq PCR PreMix (+Blue dye) (Innovagene, Changsha, China) was used for PCR amplification reaction. The PCR cycle included 95 °C for 3 min; 35 cycles of 95 °C for 30 s, gene-specific annealing at 53 °C for 30 s, and 72 °C for 2 min; and 72 °C for 10 min, then cooled to 12 °C. After being electrophoresed with 1.5% agarose gel, the target fragments were extracted with a DNA purification kit and then ligated into the pMD19-T vector at 4 °C overnight. The ligated product was converted into DH5α competent cells according to the manufacturer’s instructions and sequenced by Bio Biotech (Chengdu, China).

The position of the leptin open reading frame was analyzed with the NCBI ORF Finder (https://www.ncbi.nlm.nih.gov/orffinder (accessed on 8 December 2024)), and the signal peptide was predicted with the SignalP 6.0 Server. Protein tertiary structure prediction and domain analysis were carried out using the online tool SWISS-MODEL (https://swissmodel.expasy.org/ (accessed on 8 December 2024)). Amino acid sequence alignment was constructed with DNAMAN software version 6.0. Phylogenetic trees were built using MEGA 7 software with the Neighbor-joining method and a Bootstrap setting of 1000 based on the sequences obtained from different species.

### 4.3. Tissue Distribution and Fasting Experiments

Six juvenile Siberian sturgeons (162.05 ± 6.32 g, fasted for 24 h) were sacrificed and dissected after anaesthetization with MS-222 (100 mg/L) for nearly five minutes until the fish lost their balance reflex. Five parts of the brain (forebrain, hypothalamus, midbrain, cerebellum, and medulla oblongata) and 16 other tissues (stomach, esophagus, pyloric caecum, rectum, duodenum, valvular intestine, heart, kidneys, liver, spleen, pancreas, swim bladder, white muscle, gills, skin, and eyes) were dissected for the further analysis of *leptin* and *leptin receptor* mRNA expressions in different tissues.

In order to investigate the mRNA levels of *leptin* and *lepr* under different nutritional states, fasting and refeeding experiments were conducted. A total of 108 Siberian sturgeon (BW) were randomly distributed among 12 groups (three tanks per group, three fish per tank) and cultured for 2 weeks. After the acclimation period, five tanks were fed to satiety at one time daily (14:00) (fed groups), five tanks were fasted (fasted group), and the other two tanks were refed after 10 days of fasting (refeeding groups). The feeding groups were sampled at 1 h after feeding on days 1, 3, 6, 10, and 15, while the refeeding groups were sampled on days 10 and 15 at 1 h post-feeding. The fish were anesthetized with MS222 (100 mg/L), sacrificed, and then the liver and hypothalamus were quickly collected. Total RNA was extracted and reverse transcribed following the same protocol described in the qRT-PCR section.

### 4.4. Prokaryotic Expression of Leptin Recombinant Protein

Based on the complete coding sequence of mature protein (leptin-yf, leptin-yr, Table 1), specific primers were designed. The cDNA from Siberian sturgeon liver tissue was used as the template for amplifying the mature leptin protein of Siberian sturgeon. The PCR product was verified by sequencing after being purified using the DNA Purification Kit (Tiangen, Beijing, China). The leptin coding sequence was then inserted into the pET32a vector using T4 ligase (TransGen, Beijing, China). After transforming the recombinant plasmid of leptin-pET32a in the *E. coli* DH5α, the positive colonies were sequenced to confirm the correct sequence.

For recombinant protein expression, the leptin-pET32a plasmid was transformed into the Transetta (DE3) Cell (TransGen, China) strains of *E. coli*. The transformed strains were cultured at 37 °C with shaking at 200 rpm in 4 L of LB medium containing ampicillin (10 μg/mL). When the absorbance of medium at 600 nm reached 0.6, IPTG was added to a final concentration of 1.0 mM. After 8 h of induction, the bacterial sediment was collected by centrifugation and washed twice with PBS. The binding buffer (8 M urea, 10 mM imidazole) was used to balance the ProteinIso^®^ Ni-NTA Resin agarose column (TransGen, China). The expressed protein solution was loaded onto the column, washed with 8 M urea wash buffer (20 mM imidazole), and eluted with 8 M urea elution buffers (200 mM imidazole). The eluate product was dialyzed in PBS for 12 h and concentrated using an ultrafiltration tube. Protein concentration was measured using a BCA Kit (TransGen, China), and protein purity was analyzed by SDS-PAGE and Western blotting. The stained gels were visualized and photographed using a Gel Doc XR+ chemiluminescence gel imaging system (Bio-Rad, Hercules, CA, USA).

### 4.5. Food Intake Determination

The effect of the Ssleptin protein on feeding was studied after acute intraperitoneal (i.p.) administration. Four groups of Siberian sturgeon (49.52 ± 9.62 g) were i.p. injected with PBS or 30 ng/g, 100 ng/g, or 300 ng/g BW Ssleptin, respectively. The doses of Ssleptin were designed according to previous studies on goldfish [2] and mandarin fish [6] with slight modifications. After injection, the fish were returned to their tanks for recovery. Pre-weighed feed was provided at 14:00, and then the residual feed was collected at 1 h (15:00), 3 h (17:00), and 6 h (20:00) post-feeding. The residual feed was dried at 65 °C for 24 h and then weighed for the food intake measurement.

### 4.6. Regulation of Ssleptin on the Expression of Appetite Related Genes

To explore the mechanism by which leptin regulates food intake in Siberian sturgeon, this present study examined the expression of appetite-related genes following intraperitoneal injection of Ssleptin. Eighteen Siberian sturgeons (48.91 ± 5.29 g) were randomly divided into two experimental groups, with three tanks (three fish per tank) in each group. One group was i.p. injected with Ssleptin (100 ng/g BW), and the other was i.p. injected with PBS as a control. One hour after treatment, the fish were anesthetized with MS222 (100 mg/L) and sacrificed. Tissues, including the hypothalamus, liver, and valvular intestine, were collected and stored at −80 °C for gene expression analysis. The mRNA expression of *pomc*, *cart*, *mch*, *insulin*, *npy*, *agrp*, and *orexin* in the hypothalamus; *resistin* and *leptin* in the liver; and *pyy* and *cck* in the valvular intestine were analyzed with real-time PCR.

### 4.7. Real-Time Quantitative PCR Analysis

RNA was extracted from tissues by an RNA Extraction Kit (Foregene, Chengdu, China). The integrity of the RNA was confirmed by electrophoresis, while the purity and concentrations were measured by a NanoDrop 2000c. The cDNA was synthesized through the RT Reagent Kit (Takara, Dalian, China) and amplified using SYBR Premix Ex TapTMII (TaKaRa). The primers used for qPCR detection are shown in Table 1 and β-actin was chosen as a housekeeping gene for the normalization of the target gene expression. The qPCR protocol was performed as follows: 95 °C for 3 min, then 95 °C for 10 s, annealing for 30 s, denatured annealing for 39 cycles, extended at 95 °C for 5 s, and heated up to 95 °C for 5 s. The relative mRNA expression levels of target genes were analyzed with the relative Ct method after being normalized with the housekeeping gene.

### 4.8. SDS-PAGE and Western Blotting

The protein samples were mixed with 5 × SDS PAGE loading buffer and denatured in boiling water for 5 min. The supernatant was collected after centrifugation at 12,000 rpm for 3 min. The protein, nearly 15 μg, was electrophoresed on a 10% SDS-PAGE at 180 V for 30 min and then quickly transferred to a PVDF membrane under 100 V for 20 min. After washing three times with 1 × TBST, the PVDF membrane was blocked with 5% defatted milk at room temperature for 1 h. The PVDF membrane was incubated with primary antibodies, namely anti-His mouse monoclonal antibody (HT501-01, Transgene, Beijing, China; 1:1000), at 4 °C overnight. The protein was detected using anti-mouse IgG(H+L) HRP-conjugate second antibody (HS201-01, Transgene, Beijing, China; 1:5000) and visualized with ECL in E-Blot Xli Western Blot.

### 4.9. Statistical Analysis

All data were shown as mean ± S.E.M and analyzed using SPSS 23.0 software (IBM Inc., New York, NY, USA). The data were analyzed using Student’s *t*-test between two groups or one-way ANOVA followed by Duncan’s test for multiple groups. When the data did not meet the assumptions of normality and homogeneity, the non-parametric tests, including Mann–Whitney or Kruskal–Wallis tests, were used to analyze the significance. *p*-value < 0.05 was considered to be a significant difference.

## 5. Conclusions

This study successfully cloned the *leptin* gene from Siberian sturgeon, revealing its low conservation compared to other teleosts and mammals. In juvenile Siberian sturgeon, the expression of *leptin* mRNA was high in the liver while *lepr* was predominantly expressed in the hypothalamus. The expression levels of *leptin* and *lepr* in the hypothalamus and liver were dynamically altered by fasting and refeeding conditions. Recombinant Ssleptin protein was effectively produced using *E. coli*, and peripheral injection experiments demonstrated its acute inhibitory effect on feed intake in Siberian sturgeon. The mechanism by which Ssleptin regulates appetite involves the inhibition of orexigenic genes (*agrp*, *npy*, *ghrelin*, and *orexin*) as well as the stimulation of anorexigenic genes (*pomc*, *mch*, *insulin*, *resistin*, *pyy*, and *cck*). These findings strongly support the role of leptin as an anorexigenic factor in regulating the appetite of Siberian sturgeon. However, this study only detected the effect of Ssleptin on the expression of signaling factors at the mRNA level, which might not accurately reflect the activation or inhibition of the signaling pathway. Future studies will focus on developing antibodies against Ssleptin using the recombinant protein to analyze its expression levels in circulation and various tissues, thereby enhancing our understanding of the functions of leptin in fishes.

## Figures and Tables

**Table 1:** Primers used in present study.

Primers	Sequence (5′-3′)	Usage
*leptin*-f	GAATGAACTATCCAATTGTACCCC	Clone
*leptin*-r	CTCAGCATTTCTTTAGTTGATCCA	
*lepr*-f	CTGATTTTCAACCTCCCACA	
*lepr*-r	GAGCAGCCTACATACTTCTTCTT	
*leptin*-yf	CGCGCGGAATTCTGGCCTGTTCCAGTTGATAAA	Recombinant protein
*leptin*-yr	CCCCCCAAGCTTGCATTTCTTTAGTTGATCCAAGTTT	
*leptin*-qf	TCCTCCAGTGATAAAGCCCT	RT-qPCR
*leptin*-qr	ATACTGCCAGCGACCGAAT	
*lepr*-qf	CTGCTTGTGACGCTTGC	
*lepr*-qr	AGGTTTCCGATGGTTTCT	
*npy*-qf	GCTGGCTACCGTGGCTTTC	
*npy*-qr	GACTGGACCTCTTCCCATACCT	
*agrp*-qf	AGGCTGTGCGTCTCAGTGTC	
*agrp*-qr	GAATCGGAAGTCCTGTATCGG	
*orexin*-qf	GCTCCTGGTATGTGCCCT	
*orexin*-qr	GGGTTCGGTCTCCACAGT	
*ghrelin*-qf	CCAAGGTGACACGTCGAGATTC	
*ghrelin*-qr	GCTGTCCTTCTTGGCACTTG	
*cart*-qf	CGACTGTGGTTGAGAGCCG	
*cart*-qr	GACAGTCACACAACTTGCCGAT	
*pomc*-qf	AGCACCACCCTTAGCGTTCT	
*pomc*-qr	ACCTCTTGTCATCCCGCCT	
*mch*-qf	AACAGACACCTGCCTTAC	
*mch*-qr	AACAGACACCTGCCTTAC	
*insulin*-qf	CTGCTTGCTTTGCTTGTCTT	
*insulin*-qr	CTTCATTTTGTTGGGAGTGT	
*resistin*-qf	TAGAGGGAGCCTGGTGGATT	
*resistin*-qr	AGGTCTGGTCATTGCGGATA	
*cck*-qf	GAGGGTAGTCCTGTAGCATCTGA	
*cck*-qr	TTCTACCAGACGAGCCTTTCC	
*pyy*-qf	AGGCAGAGGTATGGCAAGCG	
*pyy*-qr	GGAGGGTCAGGAGACGGGAT	
*β-actin*-qf	GTTGGTATGGGACAGAAGGACA	
*β-actin*-qr	CCAGTTGGTAACAATGCCGT	

## Data Availability

All data presented in this study are available within the article.

## Supplementary Materials

The supporting information can be downloaded at: https://www.mdpi.com/article/10.3390/ijms26051968/s1.
