# Supplementary material for: Characterization of Leptin and Leptin Receptor Gene in the Siberian Sturgeon (Acipenser baerii): Molecular Cloning, Tissue Distribution, and Its Involvement in Feeding Regulation
Source: Int J Mol Sci. 2025 Feb 25;26(5):1968. doi: 10.3390/ijms26051968 (PMC11900199; doi:10.3390/ijms26051968)
Supplement: Supplementary file 1 [file ijms-26-01968-s001.zip › ijms-3394152-supplementary.pdf]

Supplementary Figure. S1.The sequence of Siberian sturgeon *leptin receptor*.

TGAGCAGCCTACATACTTCTTCTTATAATTGCTTTTCTATCAGCTGTTCTGCTTGTGACGCTTGCAATCTCCCAA
CACCAAATGAAGAAGCTGGTTTGGAAAGATGTGCCTAATCCTAATAATTGCTCCTGGGCTCAAGGAGTGGA
CTTTAAAAAGGCAGAAACCATCGGAAACCTCTTTAAGCACCACGAGAGGCTGACATCTGGCCCGCTTCTCTT
GGAATCAGAAAGAATTTCCGAAGCTGTAATAGTTGAGAAAATGAAACAAAGTGTGGAGGAGAAGGAGACC
AGAGTTGCCTTGGATATCTCACTGAGTCAAAGTCATGAACCGCAGCAGGATTCCCCTCCTCCTGAGCAACAC
AGCCACATTGAAGAAGAGGAGAGGGAGCCGGTGGTCTACCCAGACAGTTCAGGAGAGCCCAAAATAGAAT
ATGCCACCATCCTGCCCAGCGATCTGTACAAACAGCAGAAAAGCATCAGCAGCTCCTCCGACGAGGGGAAC
TTCTCCTGCTAACAATTCGGATATTTCTGGGTCCTTCCCCAACAATCTGTGGGAGGTTGAAAATCAG
